# Nutrient-supplemented propagation of *Saccharomyces cerevisiae* improves its lignocellulose fermentation ability

**DOI:** 10.1186/s13568-020-01070-y

**Published:** 2020-08-28

**Authors:** Marlous van Dijk, Friederike Mierke, Yvonne Nygård, Lisbeth Olsson

**Affiliations:** Dept. Biology and Bioengineering, Division of Industrial Biotechnology, Chalmers University of Technology, Kemivägen 10, 412 96 Göteborg, Sweden

**Keywords:** Corn cob hydrolysate, Wheat straw hydrolysate, Inhibitor tolerance, Vitamins, Nitrogen source, Trace metals, Industrial *Saccharomyces cerevisiae* strains

## Abstract

Propagation conditions have been shown to be of considerable importance for the fermentation ability of *Saccharomyces cerevisiae*. The limited tolerance of yeast to inhibitors present in lignocellulosic hydrolysates is a major challenge in second-generation bioethanol production. We have investigated the hypothesis that the addition of nutrients during propagation leads to yeast cultures with improved ability to subsequently ferment lignocellulosic materials. This hypothesis was tested with and without short-term adaptation to wheat straw or corn stover hydrolysates during propagation of the yeast. The study was performed using the industrial xylose-fermenting *S. cerevisiae* strain CR01. Adding a mixture of pyridoxine, thiamine, and biotin to unadapted propagation cultures improved cell growth and ethanol yields during fermentation in wheat straw hydrolysate from 0.04 g g^−1^ to 0.19 g g^−1^ and in corn stover hydrolysate from 0.02 g g^−1^ to 0.08 g g^−1^. The combination of short–term adaptation and supplementation with the vitamin mixture during propagation led to ethanol yields of 0.43 g g^−1^ in wheat straw hydrolysate fermentation and 0.41 g g^−1^ in corn stover hydrolysate fermentation. These ethanol yields were improved compared to ethanol yields from cultures that were solely short-term adapted (0.37 and 0.33 g g^−1^). Supplementing the propagation medium with nutrients in combination with short-term adaptation was thus demonstrated to be a promising strategy to improve the efficiency of industrial lignocellulosic fermentation.

## Introduction

The limited tolerance of *Saccharomyces cerevisiae* to inhibitors present in lignocellulosic hydrolysates is a major challenge in second-generation bioethanol production (Palmqvist and Hahn-Hägerdal 2000; Hemansi et al. 2019). Synergistic effects have been reported between lignocellulosic inhibitors (Oliva et al. 2006; Ding et al. 2011), as well as between lignocellulosic inhibitors and process conditions prevailing during lignocellulosic fermentations (e.g. high osmolarity, poor mixing, etc.), as reviewed by Piotrowski et al. (2014). Among the strategies used to counteract inhibitory effects, the addition of inorganic nutrients to lignocellulosic fermentation medium has been shown to improve the fermentation performance (Jørgensen 2009; Xiros and Olsson 2014; Kelbert et al. 2015). The present study takes another approach by investigating the effects of nutrient addition to the propagation on lignocellulose fermentation performance of *S. cerevisiae.* It also investigates whether the efficiency could be improved further by combining it with short-term adaptation.

### Strategies for improved lignocellulose fermentation

Despite the existence of complete biosynthetic pathways in the *Saccharomyces cerevisiae* genome to produce B vitamins (Perli et al. 2020), most chemically defined media for yeast cultivations include these vitamins to support faster cell growth (Verduyn et al. 1992). Additionally, supplementation of nutrients such as biotin, magnesium, or zinc to the fermentation medium have been reported to improve ethanol tolerance, and thus ethanol productivity in media without lignocellulose hydrolysates (Dombek and Ingram 1986; Winter et al. 1989; Alfenore et al. 2002; Zhao et al. 2009). The addition of manganese in yeast fermentation has been reported to improve xylose consumption under acetic acid stress (Ko et al. 2016), while the addition of complex nutrients to high-gravity lignocellulose fermentation has been shown to improve the fermentation performance (Xiros and Olsson 2014). Nutrient supplementation to the lignocellulose fermentation medium directly thus appears to improve the fermentation efficiency. However, information is lacking on the effects of addition of nutrients to the propagation media and how such additions change the cells capacity to ferment lignocellulose hydrolysates.

Short-term adaptation is achieved by propagating yeast in dilute lignocellulosic hydrolysate medium. Physiological parameters such as viability, biomass yield, and fermentation capacity have been reported to increase during fermentation following short-term adaptation compared to cultures propagated in a medium without hydrolysate (Alkasrawi et al. 2006; Nielsen et al. 2015; Zhang et al. Zhang et al. 2019; van Dijk et al. 2019). Alternatively, evolutionary engineering, or long-term adaptation, of yeast strains has been shown to be successful in increasing the efficiency of fermentation through increased inhibitor tolerance (Marti´n and Jönsson 2003; Tomás-Pejó et al. 2010; Brandt et al. 2019). However, hydrolysate composition, and thus inhibitor abundance, varies depending on the feedstock (Klinke et al. 2004; Almeida et al. 2007), seasonality (Bunnell et al. 2013; Greenhalf et al. 2013), and method of substrate pretreatment (Chundawat et al. 2010). As inhibitor profiles vary, there is a risk that yeast strains will be evolved for a specific inhibitor profile, leading to a sub-optimal efficiency for others.

The aim of the present study was to investigate the effects of nutrient supplementation of various vitamins, trace metals, and nitrogen sources during the propagation of the industrial xylose-fermenting strain of *S. cerevisiae*, CR01, with and without the presence of lignocellulosic hydrolysate (short-term adaptation). The cells that were propagated under different conditions were evaluated on their efficiency to ferment lignocellulosic hydrolysates. We hypothesized that the addition of nutrients during propagation would produce cells with improved capacity to ferment lignocellulosic hydrolysates.

## Materials and methods

### Microorganism and cultivation

The industrial strain of *S. cerevisiae* used in this study was CR01, kindly provided by Taurus Energy AB, Sweden. This strain harbors the xylose-utilization genes Xyl1 (xylose reductase) and Xyl2 (xylitol dehydrogenase) from *Pichia stipitis*, and overexpresses the endogenous XKS1 (xylulokinase) gene. This strain has also been subjected to evolutionary engineering to improve its xylose fermentation efficiency and its tolerance to lignocellulosic inhibitors. It was stored at − 80 °C in a 30% (w/w) glycerol solution.

### Seed cultivation

Before propagation, the frozen cell stock solutions were thawed and grown for 24 h in synthetic minimal medium containing 20 g L^−1^ glucose at pH 6.0. Other components were added according to Verduyn et al. (1992), with the exception of ammonium sulfate, which was replaced by 2.3 g L^−1^ urea to prevent acidification of the medium. Incubation was performed at 30 °C on an orbital shaker (IKA, Germany) at 200 rpm (orbital diameter: 20 mm) in 250 mL shake flasks with a working volume of 50 mL.

### Propagation

Aerobic propagation was performed in 250 mL shake flasks with a working volume of 50 mL. Cultures were inoculated to an OD of 0.1, incubated at 30 °C and agitated at 200 rpm in an orbital shaker. The propagation control medium consisted of 2.3 g L^−1^ urea, 3 g L^−1^ potassium phosphate, 0.5 g  L^−1^ magnesium sulfate, and 0.11 µg L^−1^ d-biotin. As a carbon source, 30 g L^−1^ glucose and 15 g L^−1^ of xylose were used. Various nutrients were added during propagation in an initial screening, including vitamins, trace metals, and different nitrogen sources, according to Table 1. Choice of nutrients and their concentrations resulted from a literature search. Certain conditions were selected and tested in an experiment where fermentation cultures were scaled-up (Table 2).

**Table 1:** Screening experimental conditions, stating concentrations of nutrients additions

Propagation	Fermentation
Experimental set A	
Control medium	Control medium
0.165 µg L^−1^ of d-biotin	Control medium
0.44 µg L^−1^ of d-biotin	Control medium
1 mg L^−1^ pyridoxine	Control medium
1 mg L^−1^ thiamine	Control medium
1 mg L^−1^ pyridoxine, 1 mg L^−1^ thiamine, and 0.44 µg L^−1^ of d-biotin	Control medium
50 mg L^−1^ zinc sulfate	Control medium
10 mg L^−1^ manganese chloride	Control medium
30 mg L^−1^ iron sulfate	Control medium
5 g L^−1^ ammonium sulfate as nitrogen source (+ phosphate buffer)	Control medium
3.44 g L^−1^ peptone as nitrogen source (+phosphate buffer)	Control medium
Control medium (+ phosphate buffer)	Control medium
Experimental set B	
Control medium	Control medium
0.165 µg L^−1^ of d-biotin	0.165 µg L^−1^ of d-biotin
0.44 µg L^−1^ of d-biotin	0.44 µg L^−1^ of d-biotin
1 mg L^−1^ pyridoxine	1 mg L^−1^ pyridoxine
1 mg L^−1^ thiamine	1 mg L^−1^ thiamine
1 mg L^−1^ pyridoxine, 1 mg L^−1^ thiamine, and 0.44 µg L^−1^ of d-biotin	1 mg L^−1^ pyridoxine, 1 mg L^−1^ thiamine, and 0.44 µg L^−1^ of d-biotin
50 mg L^−1^ zinc sulfate	50 mg L^−1^ zinc sulfate
10 mg L^−1^ manganese chloride	10 mg L^−1^ manganese chloride
30 mg L^−1^ iron sulfate	30 mg L^−1^ iron sulfate
5 g L^−1^ ammonium sulfate as nitrogen source (+ phosphate buffer)	5 g L^−1^ ammonium sulfate as nitrogen source (+ phosphate buffer)
3.44 g L^−1^ peptone as nitrogen source (+ phosphate buffer)	3.44 g L^−1^ peptone as nitrogen source (+ phosphate buffer)
Control medium (+ phosphate buffer)	Control medium (+ phosphate buffer)

Propagation was performed in shake flasks and fermentation in microbioreactors. Each row indicates the combination of the propagation and fermentation medium used. Unless otherwise stated nutrients were added to the control medium

**Table 2:** Scaled-up experimental conditions, stating concentrations of nutrients additions

Propagation	Fermentation
Control medium	Control medium
1 mg L^−1^ pyridoxine	Control medium
1 mg L^−1^ thiamine	Control medium
1 mg L^−1^ pyridoxine, 1 mg L^−1^ thiamine, and 0.44 µg L^−1^ of d-biotin	Control medium

Propagation and fermentation were both performed in shake flasks. Each row indicates the combination of the propagation and fermentation medium used. Unless otherwise stated nutrients were added to the control medium

Additionally, all propagation conditions in Table 1 and Table 2 were tested in the presence of 40% (w/w) wheat straw hydrolysate (WSH), or 20% (w/w) corn stover hydrolysate (CSH), as these concentrations were found to result in efficient short-term adaptation. The control media contained the same glucose and xylose concentrations as the hydrolysates in the corresponding adaptation media. After propagation, cells were harvested, washed in a 9 g L^−1^ sodium chloride solution, and resuspended in fermentation medium.

### Fermentation

In the initial screening fermentation cultures with a working volume of 1 mL were carried out using 48-well FlowerPlates in a Biolector I (m2p–labs GmbH, Germany). The microbioreactor fermentations were performed in the anaerobic chamber of the device, at 30 °C, while being agitated at 800 rpm and flushed with nitrogen gas. Scattered light was used as a measure of cell growth (arbitrary units), and values were recorded every 20 min. Fermentation was continued for 48 h, at which time samples were taken for OD and HPLC measurements.

Propagation conditions that showed to affect fermentation efficiency were selected from the screening results and scaled-up to be performed in 500 mL screw-top shake flasks (Duran, Germany) with a working volume of 200 mL. The shake flasks were incubated at 30 °C and agitated at 150 rpm in an orbital shaker. A one-way valve was connected to the cap (Eppendorf, Germany) to allow for carbon dioxide release, while another connection allowed for sterile sampling through a swabable valve without opening the shake flask. Weight loss due to carbon dioxide release was monitored and used to determine the progress of the fermentation.

The fermentation control medium consisted of 80% (w/w) WSH or 70% (w/w) CSH, supplemented with 2.3 g L^−1^ urea, 3 g L^−1^ potassium phosphate, 0.5 g L^−1^ magnesium sulfate, and 0.11 µg L^−1^ d-biotin. Each fermentation was inoculated to an OD of 1.0. During the initial screening, in experimental set A (Table 1), nutrient additions to the propagation were investigated followed by fermentation in the control medium (no additional nutrients). In experimental set B (Table 1) nutrient additions to both the propagation and fermentation were investigated. In the scaled-up experiments, fermentations were only performed without additional nutrient additions (Table 2).

### Raw material, pretreatment, and enzymatic hydrolysis

Hydrolysates from two different lignocellulosic materials were used: wheat straw and corn stover. The wheat straw and corn stover were impregnated in a 0.2% (w/w) solution of sulfuric acid for at least 1 h. The resulting material was filter pressed to a dry matter content of 40% (w/w), and subsequently incubated in a steam pretreatment unit for 10 min at 190 °C (wheat straw) or 200 °C (corn stover), as described earlier (Linde et al. 2008).

In order to obtain liquid hydrolysates, the steam-pretreated slurries were first diluted to a water insoluble solids content of 10% (w/w). The enzyme cocktail Cellic Ctec 2 (Novozymes, Denmark) was added to the steam-pretreated wheat straw slurry at a concentration of 10 FPU g_WIS_^−1^. Enzymatic hydrolysis was performed in a stirred reactor at 45 °C, pH 4.8. The sugars and degradation products in the resulting WSH and CSH were determined (Table 3).

**Table 3:** Composition of the liquid fractions of the wheat straw and corn stover hydrolysate after enzymatic hydrolysis

	Wheat straw hydrolysate (g L^−1^)	Corn stover hydrolysate (g L^−1^)
Glucose	75.3	68.0
Xylose	38.2	24.2
Arabinose	6.4	5.0
Cellobiose	8.6	6.4
Formic acid	1.6	2.0
Acetic acid	5.1	5.2
Levulinic acid	0.0	0.1
HMF	0.1	0.2
Furfural	2.8	3.0

### Analytical methods

#### Cell density measurements

The optical density (OD) was determined at 600 nm using a Genesys 20 spectrophotometer (Thermo Scientific, USA). The value measured from filtered samples was subtracted to compensate for the background of the medium. The cell dry weight (CDW) was determined by filtering appropriate volumes (containing a minimum of 10 mg_CDW_ and a maximum of 40 mg_CDW_ on the filter) of cell culture through a pre-dried and weighed 0.45 µm polyethersulfone membrane (Sartorius, Germany). The filters containing samples were washed with deionized water and dried again in a microwave oven at a power output of 385 W for 15 min, before final weighing.

#### Metabolite and inhibitor analysis

The concentrations of extracellular metabolites, sugars, and inhibitors were determined with HPLC, using a refractive index detector (Jasco, Italy). Measurements were performed on filtered samples (0.2 µm nylon membrane filters, VWR, USA). Glucose, xylose, arabinose, formic acid, acetic acid, 5-(hydroxymethyl) and furfural (HMF), were separated using a Rezex ROA-Organic Acid H^+^ column at a flow rate of 0.8 mL min^−1^, at 80 °C, using 5 mM sulfuric acid solution as eluent.

## Results

The influence of different nutrient additions to the propagation and fermentation media on lignocellulose fermentation performance was first tested using a microbioreactor system. This allowed for parallel screening of many conditions. The results obtained from these tests, i.e. the ethanol yield and xylose consumption, were used for informed design of the subsequent fermentations in shake flasks.

### Screening of vitamin supplementation during cell propagation

**Fig. 1:**
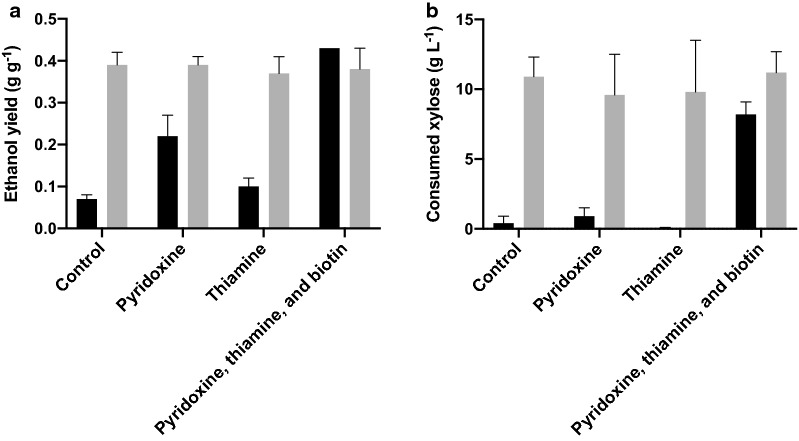
Influence of vitamin supplementation during propagation of *Saccharomyces cerevisiae* strain CR01 on fermentation performance in microbioreactors using a medium containing 80% (w/w) wheat straw hydrolysate. **a** The ethanol yield on total sugars, and **b** consumed xylose after 48 h. Results of unadapted cultures are shown in black and results of adapted cultures are shown in grey

In the screening experiments pyridoxine, thiamine, and biotin were added to the propagation media both separately and combined. Results showed that the addition of a mixture of pyridoxine, thiamine and biotin, during propagation led to an increase in the ethanol yield on total sugars during WSH fermentation (0.43 g g^−1^), compared to non-supplemented, unadapted cultures (0.07 g g^−1^, Fig. 1a). Pyridoxine addition increased the ethanol yield (0.22 g g^−1^, Fig. 1a), whereas thiamine supplementation did not lead to any increase in ethanol yield when using unadapted cultures (Fig. 1a), compared to their respective control cultures. In the case of adapted cells, no clear increase in ethanol yield during WSH fermentation was observed when the propagation culture was supplemented with any of the vitamins or the mixture (Fig. 1a).

No improvements in xylose consumption were seen when the vitamins were added separately during the propagation. However, supplementation with the mixture of vitamins led to improved xylose utilization during WSH fermentation (8.2 g L^−1^, Fig. 1b), compared to the control medium (0.4 g L^−1^, Fig. 1b). No improvement was seen in xylose consumption when vitamins were added to the adapted cultures (Fig. 1b). The addition of biotin alone during propagation (0.165 and 0.44 µg L^−1^) did not lead to a clear improvement in fermentation performance in either unadapted or adapted cultures, compared to when the yeast was propagated without supplementation (data not shown).

**Fig. 2:**
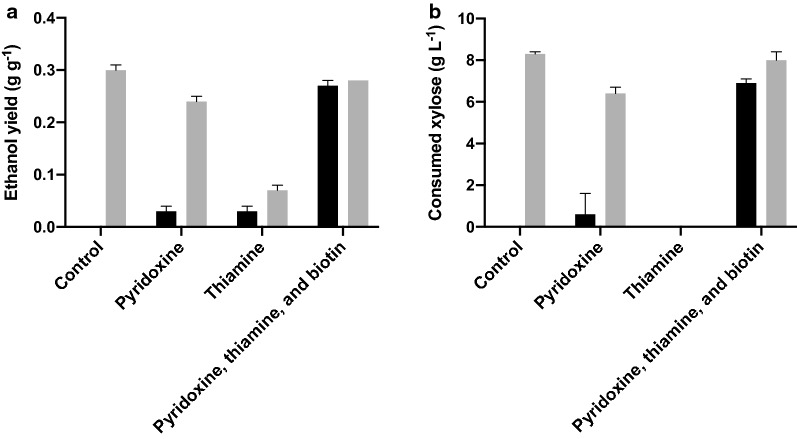
Influence of vitamin supplementation during propagation of *Saccharomyces cerevisiae* strain CR01 on fermentation performance in microbioreactors using a medium containing 70% (w/w) corn stover hydrolysate. **a** The ethanol yield on total sugars, and **b** consumed xylose after 48 h. Results of unadapted cultures are shown in black and results of adapted cultures are shown in grey

When CSH was used as fermentation medium, no growth was observed in unadapted cultures propagated in the control medium, with pyridoxine, or with thiamine supplementation, or in adapted cultures with thiamine supplementation, (data not shown). Under these conditions, the ethanol yields were between 0.00 and 0.07 g g^−1^ (Fig. 2a). Only minor xylose consumption was observed in these cultures after 48 h (Fig. 2b).

Under the other conditions investigated: unadapted cultures supplemented with the vitamin mixture, and adapted cultures grown in the control medium, with pyridoxine, and with the vitamin mixture, the ethanol yields were between 0.24 g g^−1^ and 0.30 g g^−1^ (Fig. 2a). As in the case of WSH, the ethanol yields from the fermentation of CSH were not improved using the adapted cells when supplemented with the vitamin mixture (0.28 g g^−1^), compared to the control medium (0.30 g g^−1^ Fig. 2b).

### Screening of different nitrogen sources during propagation

Urea was used as the nitrogen source in the control medium. In order to investigate the influence of the nitrogen source on the short-term adaptation effect, urea was replaced with ammonium sulfate or peptone during propagation. WSH fermentation growth curves obtained from the studies in microbioreactors showed that cultures in which urea was used as the nitrogen source grew better than cultures with ammonium (data not shown). Urea and peptone supplementation resulted in similar ethanol yields for both adapted and unadapted cells (0.36–0.37 g g^−1^, Table 4). The ethanol yield achieved with adapted cells when ammonium was used as the nitrogen source was lower than the yields obtained using the other nitrogen sources (0.21 g g^−1^, Table 4), which is consistent with the finding that all the glucose was not consumed after 48 h (data not shown). The trends in xylose consumption when urea and peptone were used as nitrogen sources mirrored those of the ethanol yields. Higher xylose consumption was observed when using urea as the nitrogen source (15 L^−1^ for unadapted and 14 g L^−1^ for adapted cells), compared to ammonium (11 g L^−1^ for unadapted and 3 g L^−1^ for adapted cells), or peptone (12 g L^−1^ for unadapted and 13 g L^−1^ for adapted cells, Table 4).

**Table 4:** Influence of different nitrogen sources in the propagation medium on the ethanol yield on total sugars and xylose consumption of CR01 during fermentation of wheat straw and corn stover hydrolysates

	Nitrogen source	Wheat straw hydrolysate		Corn stover hydrolysate	
		Ethanol yield (g g^−1^)	Consumed xylose (g L^−1^)	Ethanol yield (g g^−1^)	Consumed xylose (g L^−1^)
No adaptation	Urea^a^	0.36 ± 0.02	15.1 ± 0.4	0.35 ± 0.03	7.4 ± 1.0
	Ammonium^b^	0.35 ± 0.01	11.4 ± 0.5	0.29 ± 0.00	4.9 ± 0.7
	Peptone^c^	0.35 ± 0.01	12.6 ± 0.6	0.20 ± 0.02	2.2 ± 0.8
Adaptation	Urea^a^	0.36 ± 0.00	14.7 ± 0.4	0.36 ± 0.02	9.5 ± 0.6
	Ammonium^b^	0.21 ± 0.01	3.3 ± 0.6	0.34 ± 0.01	8.3 ± 0.3
	Peptone^c^	0.37 ± 0.00	13.8 ± 0.3	0.27 ± 0.01	2.9 ± 0.3

^a^2.3 g L^−1^; ^b^5.0 g L^−1^; ^c^3.4 g L^−1^

In CSH, ammonium-supplemented cultures of CR01 performed similarly to those supplemented with urea (Table 4). In contrast, cultures supplemented with peptone showed lower ethanol yields (0.20 g g^−1^ for unadapted and 0.27 g g^−1^ for adapted CR01, Table 4), and xylose consumption (2.2 g L^−1^ for unadapted and 2.9 g L^−1^ for adapted CR01, Table 4), than with the other two nitrogen sources.

### Screening of trace metal supplementation during cell propagation

Zinc, manganese, or iron were added to propagation media to investigate their effect on lignocellulose fermentation performance. The addition of trace metals to the propagation of CR01 in the medium without hydrolysate added led to impaired growth (OD_48h_ reached between 0.2 and 1.5, Additional file 1: Table S1). Fermentation media could not be inoculated with cells grown under these conditions due to insufficient growth. The addition of trace metals to the adapted cultures led to similar growth to that seen with vitamin additions (OD_48h_ reached between 3.3 and 4.1, Additional file 1: Table S1). No significant differences in growth, ethanol yield, or xylose consumption during fermentation by adapted CR01 grown in the presence of trace metals were seen, compared to the non-supplemented conditions, in either WSH or CSH (data not shown). As no improvements were seen, trace metals were not further investigated.

### Screening of nutrient supplementation during both propagation and fermentation

Thus far, only results from cultures with nutrients supplemented during propagation have been described (experimental set A, Table 1). A screening was also carried out where nutrients were supplementing to both propagation and fermentation (experimental set B, Table 1). No improved cell growth, ethanol yield or xylose consumption was observed when experimental set B was compared to set A. We therefore concluded that cells that have been propagated under conditions that allowed them to maintain their capacity to efficiently ferment lignocellulosic hydrolysate without the need for further nutrient addition during said fermentation.

### Vitamin addition during propagation in shake flasks

After the initial experiments in microbioreactors, fermentation was scaled up to be carried out in shake flasks with a working volume of 200 mL. Based on the results of screening in microbioreactors, a number of conditions were chosen for further investigation: the control medium, pyridoxine, thiamine, and the vitamin mixture. Similar trends were observed in the shake flask fermentation (i.e. ethanol yield, xylose consumption, and furfural detoxification) of both WSH and CSH. Unadapted CR01 cultures to which the vitamin mixture had been added showed growth in both WSH and CSH (final OD 2.4 and 4.7, respectively), as well as furfural depletion. No xylose consumption was observed under these conditions and the ethanol yields reached were 0.19 g g^−1^ (WSH) and 0.08 g g^−1^ (CSH, Table 5). Unadapted CR01 cultures supplemented only with thiamine, only with pyridoxine and those grown in the control medium, showed no growth. The ethanol yields were below 0.04 g g^−1^, and no xylose was consumed within 48 h, for either of the hydrolysates (Table 5). Furthermore, furfural was still present in the medium at 48 h.

**Table 5:** Results of anaerobic fermentation of unadapted and adapted CR01 cultures with and without the addition of vitamins

Conditions	OD_600nm_	Ethanol yield (g g^−1^)	Overall specific ethanol productivity^f^ (g OD^−1^ h^−1^)	Consumed xylose (g L^−1^)	Furfural depletion
Propagation medium					
Wheat straw hydrolysate^d^					
No adaptation					
Control	1.4 ± 0.0	0.04 ± 0.00	0.03 ± 0.00	–	No
Thiamine	1.3 ± 0.1	0.04 ± 0.01	0.05 ± 0.01	–	No
Pyridoxine	1.2 ± 0.1	0.03 ± 0.01	0.03 ± 0.01	–	No
Mixture^c^	2.4 ± 1.1	0.19 ± 0.11	0.11 ± 0.01	–	Yes
Adaptation^a^					
Control	3.9 ± 0.4	0.37 ± 0.00	0.13 ± 0.01	3.3 ± 0.5	Yes
Thiamine	3.3 ± 0.8	0.42 ± 0.01	0.13 ± 0.02	5.3 ± 0.7	Yes
Pyridoxine	3.4 ± 0.8	0.41 ± 0.00	0.13 ± 0.02	7.2 ± 0.9	Yes
Mixture^c^	3.4 ± 0.8	0.43 ± 0.01	0.13 ± 0.02	6.7 ± 0.5	Yes
Corn stover hydrolysate^e^					
No adaptation					
Control	1.5 ± 0.0	0.02 ± 0.02	0.03 ± 0.00	–	No
Thiamine	1.4 ± 0.1	0.03 ± 0.02	0.03 ± 0.02	0.3 ± 0.1	No
Pyridoxine	1.6 ± 0.1	0.01 ± 0.00	0.01 ± 0.00	0.6 ± 0.9	No
Mixture^c^	4.7 ± 0.3	0.08 ± 0.00	0.02 ± 0.00	0.5 ± 0.1	Yes
Adaptation^b^					
Control	3.8 ± 0.5	0.33 ± 0.01	0.10 ± 0.01	3.0 ± 1.0	Yes
Thiamine	1.9 ± 0.5	0.14 ± 0.02	0.09 ± 0.01	0.7 ± 0.1	Yes
Pyridoxine	4.5 ± 0.2	0.40 ± 0.01	0.09 ± 0.01	4.1 ± 1.1	Yes
Mixture^c^	5.0 ± 0.1	0.41 ± 0.01	0.10 ± 0.00	5.6 ± 0.4	Yes

^a^Cultures were propagated in medium containing 40% (w/w) wheat straw hydrolysate

^b^Cultures were propagated in medium containing 20% (w/w) corn stover hydrolysate

^c^Mixture of 1 mg L^−1^ pyridoxine, 1 mg L^−1^ thiamine, and 0.44 µg L^−1^ biotin

^d^Fermentation medium contained 80% (w/w) wheat straw hydrolysate

^e^Fermentation medium contained 70% (w/w) corn stover hydrolysate

^f^Overall specific ethanol productivity was calculated using the ethanol and OD values measured at the t_0h_ and the t_48h_ sampling points

Growth (defined as at least one doubling of the OD value) was observed in both WSH and CSH for all adapted cultures except for CSH supplemented with thiamine (OD_48h_ 3.3–5.0, Table 5). Fermentation by adapted cells that showed growth, gave xylose consumption (3.0 g L^−1^–7.2 g L^−1^) and ethanol yields (0.33 g g^−1^–0.43 ± g g^−1^) that were higher than those with unadapted cultures. Furthermore, furfural depletion was observed, whereas no depletion was seen with unadapted cultures (Table 5). Thiamine supplementation of the CSH medium resulted in impaired growth (OD_48h_ 1.9), low xylose consumption (0.7 g L^−1^), and a low ethanol yield (0.14 g g^−1^) (Table 5). The specific ethanol productivity of adapted CR01 cultures did not increase as a result of supplementation with vitamins (Table 5).

## Discussion

The role of nutrient additions during propagation has not been extensively investigated for lignocellulosic hydrolysate fermentations, and here we demonstrated the potential to improve the process by focusing on the propagation step. Earlier studies have shown that short-term adaptation during propagation can improve cellular performance significantly and here we screened different nutrients as an alternative and complementary approach to improve the propagation.

The screening of addition of different vitamins to the propagation in the present study showed that addition of a mixture of vitamins during yeast propagation improved the fermentation performance of unadapted cells, but not that of adapted cultures in both WSH and CSH. This indicates that vitamin supplementation, as well as short-term adaptation, altered the metabolic states of the cultures, allowing the cells to cope better with the harsh environment of the lignocellulosic hydrolysate medium. Vitamin synthesis, like biosynthesis, requires considerable energetic input from the cell, therefore, supplementation with vitamins could allow for re-allocation of that chemical energy towards growth and synthesis and storage of compounds that improve robustness.

Thiamine addition to the propagation medium inhibited fermentation performance in CSH medium. Growth inhibition by thiamine in *S.* *cerevisiae* cultures has previously been observed, possibly due to the depletion of intracellular pyridoxine (Nakamura et al. 1982). The reason for difference in growth inhibition by thiamine in CSH and WSH cultures observed in the present study could be due to presence of different inhibitors in the respective hydrolysates. The complex picture of the inhibitory action by individual inhibitors makes it impossible to pin-point the cause of the differences seen. The results from the vitamin screening indicated that the combination of pyridoxine, thiamine, and biotin might be required for short-term adaptation. Alternatively, the addition of the mixture of vitamins could simply make cellular resources available for inhibitor tolerance mechanisms. Pyridoxine (vitamin B_6_) was included in the experimental design as it has been shown to exhibit better antioxidant properties than vitamin C or E (Bilski et al. 2004; Mooney et al. 2009). Thiamine (vitamin B_1_) has been shown to be involved in the maintenance of redox balance under oxidative stress conditions (Wolak et al. 2014), all indicating that these vitamins may contribute to increased robustness when cells are challenged.

Furfural is known to inhibit glycolysis in yeast (Banerjee et al. 1981), and furfural detoxification has been reported to be an important coping strategy of *S.* *cerevisiae* (Liu 2011). Our results confirmed that the cultures must first detoxify the medium by depleting the furfural before growth and fermentation can commence. Cultures that had depleted furfural started to grow and produce ethanol, while the opposite was observed in all cultures that still contained furfural. Furthermore, our data shows that the addition of the vitamin mixture to the propagation improved the ability of cultures to convert furfural during the fermentation.

Devantier et al. (2005) have reported that the specific ethanol productivity remains high and constant during the exponential growth phase, but ethanol productivity decreased when the cells stopped to grow. Similarly, our results also show a correlation between cell mass increase and ethanol productivity, pointing out the importance of increase of growth ability during the fermentation.

Unadapted propagation cultures that had been supplemented with the vitamin mixture showed improved ethanol yields on total sugars when used to ferment WSH and CSH (WSH from 0.0408 g g^−1^ to 0.19 g g^−1^ and CSH from 0.0208 g g^−1^ to 0.08 g g^−1^). The ethanol yields were further improved when adaptation and supplementation of the vitamin mixture were combined, to 0.43 and 0.41 g g^−1^, respectively, compared to 0.37 and 0.33 g g^−1^, respectively for adapted cultures without vitamin addition. Regarding xylose consumption, fermentations inoculated with cultures supplemented with pyridoxine and the vitamin mixture performed better than those inoculated with thiamine-supplemented cultures, and all the vitamin-supplemented cultures performed better than the non–supplemented cultures. This indicates that although short-term adaptation during propagation improves lignocellulose fermentation performance without vitamin supplementation, nutrient supplementation during the short-term adaptation can further improve the effect. This study shows the promising potential of nutrient addition during cell propagation to improve the fermentation of lignocellulosic hydrolysates.

## Supplementary information

**Additional file 1: Table S1.** Growth during the propagation of CR01 expressed as OD_600_ values measured after 48 h. (Values given are the average of two duplicate experiments in shake flasks).

## Data Availability

Not applicable.
