# Supplementary material for: Nutrient-supplemented propagation of Saccharomyces cerevisiae improves its lignocellulose fermentation ability
Source: AMB Express. 2020 Aug 28;10:157. doi: 10.1186/s13568-020-01070-y (PMC7455642; doi:10.1186/s13568-020-01070-y)
Supplement: Supplementary file 1 — Additional file 1: Table S1. Growth during the propagation of CR01 expressed as OD600 values measured after 48 h. (Values given are the average of two duplicate experiments in shake flasks). [file 13568_2020_1070_MOESM1_ESM.docx]

# Additional file

**Table S1.** Growth during the propagation of CR01 expressed as OD_600_ values measured after 48 h. (Values given are the average of two duplicate experiments in shake flasks.)

| Condition | 0% WSH  OD_600nm_ | 40% WSH  OD_600nm_ | 0% CSH  OD_600nm_ | 20% CSH  OD_600nm_ |
| --- | --- | --- | --- | --- |
| Control | 3.0 ± 0.1 | 2.6 ± 0.1 | 2.9 ± 0.2 | 3.3 ± 0.0 |
| Buffered control | 2.8 ± 0.1 | 4.4 ± 0.4 | 5.0 ± 0.4 | 3.1 ± 0.2 |
| 2.5x Biotin | 2.4 ± 0.1 | 2.9 ± 0.3 | 3.8 ± 0.4 | 3.4 ± 0.2 |
| 5x Biotin | 2.5 ± 0.4 | 2.7 ± 0.1 | 3.2 ± 0.2 | 3.4 ± 0.0 |
| Thiamine | 3.3 ± 0.2 | 2.9 ± 0.0 | 3.2 ± 0.0 | 3.3 ± 0.6 |
| Pyridoxine | 3.7 ± 0.3 | 2.9 ± 0.2 | 3.4 ± 0.1 | 3.3 ± 0.8 |
| Vitamin mixture | 3.3 ± 0.1 | 3.1 ± 0.1 | 3.1 ± 0.0 | 3.7 ± 0.0 |
| Ammonium | 2.9 ± 0.2 | 2.5 ± 0.1 | 6.1 ± 0.1 | 3.1 ± 0.3 |
| Peptone | 8.5 ± 0.8 | 6.7 ± 0.0 | 8.2 ± 0.1 | 8.9 ± 0.4 |
| Zinc | 0.9 ± 0.7 | 3.4 ± 0.0 | 2.0 ± 0.1 | 3.3 ± 0.2 |
| Manganese | 0.2 ± 0.0 | 3.4 ± 0.1 | 0.3 ± 0.0 | 3.6 ± 0.3 |
| Iron | 1.5 ± 1.4 | 4.1 ± 0.2 | 3.3 ± 0.0 | 3.6 ± 0.0 |
